# Investigating the Perceptions of Primary Care Dietitians on the Potential for Information Technology in the Workplace: Qualitative Study

**DOI:** 10.2196/jmir.9568

**Published:** 2018-10-15

**Authors:** Aimee Jones, Lana J Mitchell, Rochelle O'Connor, Megan E Rollo, Katherine Slater, Lauren T Williams, Lauren Ball

**Affiliations:** 1 School of Allied Health Sciences Griffith University Gold Coast Australia; 2 Menzies Health Institute Queensland Griffith University Gold Coast Australia; 3 Priority Research Centre for Physical Activity and Nutrition University of Newcastle Newcastle Australia

**Keywords:** dietetics, information technology, mobile phone, primary health care, private practice

## Abstract

**Background:**

Chronic diseases are the leading cause of morbidity and mortality worldwide. The primary health care setting is an effective avenue for the management and prevention of chronic diseases. Dietitians working in this setting assist with the management of modifiable risk factors of chronic diseases. However, health care professionals report challenges in providing care in this setting because of time and financial constraints. Information technology offers the potential to improve health care quality, safety, efficiency, and cost-efficiency, but there exists limited understanding of dietitians’ application of technology in this setting.

**Objective:**

The objective of this study was to explore the perceptions of primary care dietitians about using information technology in their workplace.

**Methods:**

We recruited 20 Australian primary care dietitians using purposive and snowball sampling for semistructured telephonic interviews. Interview questions aimed to gain an understanding of dietitians’ perceptions about sharing patient outcomes through a national database and the benefits, disadvantages, feasibility, and barriers of using information technology. Interviews were audiorecorded, transcribed verbatim, and thematically analyzed for emerging themes and subthemes. Finally, the technologies used by participants were collated by name and researched for their key attributes.

**Results:**

The following 4 distinct themes emerged from the data: information technology improving the efficiency of practice tasks, experiencing barriers to using information technology in practice, information technology enhancing outcomes through education and monitoring, and information technology for sharing information with others. Participants identified several advantages and disadvantages of using technology and expressed willingness to share patient outcomes using a Web-based database.

**Conclusions:**

This study suggests that information technology is perceived to have benefits to dietitians and patients in primary health care. However, to achieve the optimal benefit, support is required to overcome barriers to integrate information technology into practice better. Further development of patient management systems and standardized Web-based data collection systems are needed to support better usage by dietitians.

## Introduction

Chronic diseases are a leading cause of morbidity and mortality worldwide [1]. The occurrence of chronic diseases is associated with preventable risk factors such as high blood pressure, high blood cholesterol, and overweight or obesity [1]. Dietitians are uniquely qualified to apply the science of nutrition to the feeding and education of individuals or groups with chronic disease risk factors [2]. Primary health care is an effective avenue for the management and prevention of chronic diseases [3]. However, primary health care professionals, including dietitians, report challenges in providing effective care because of time and financial constraints under profession-specific requirements [4-7]. Australian dietitians report restricting the consultation frequency and length because of insufficient funding under the Australian Medicare scheme [4,8]. Such “abbreviated care” is of concern for patient health outcomes [4,8]. Thus, tools to increase the efficiency and effectiveness of practice are important for dietitians working in this sector.

Information technology is recognized as supporting improvements in the health care quality, safety, efficiency, and cost efficiency [7,9,10]. Moreover, information technology facilitates the increased productivity in health care by reducing the time required to complete tasks [11-13]. In the context of the dietetic practice, dietitians can use information technology, such as electronic health records, in consultations for automatic calculations (ie, anthropometry and dietary intake), electronic database storage of patient information, and prepopulated prompting within chart entries (ie, diagnosis prompts based on assessment) [11]. Electronic health records are longitudinal records of patient health information produced from encounters in any health care delivery setting [14]; these can support efficiency, better collaboration between health professions, and provide potential to measure digital health outcomes and inform research to promote better patient outcomes [15,16]. In addition, mobile technology has been used to support dietetic care outside of consultations, such as for motivational reminders and feedback for patients [17-19] and conduct remote dietary tracking [20]. Strategies that support dietitians to utilize information technology are, therefore, likely to have a positive impact on the dietetic practice.

Despite the potential contribution to effective practice, there is limited understanding about the way dietitians use information technology in practice. The opportunity for practice improvements using information technology is particularly relevant to the primary care sector in which >25% of Australian dietitians’ work [21]. Although a body of literature exists investigating dietitians’ use of a mobile app [22,23], a better understanding of the broader use of information technology is needed. Moreover, greater use of information technology, such as electronic health records, by primary care dietitians may facilitate national-scale reporting of patient outcomes to inform policy making and dietetic advocacy. Therefore, this study aims to investigate the perceptions and experiences of Australian primary care dietitians about using information technology in practice.

## Methods

### Research Overview

This study was underpinned by a descriptive exploratory approach [24] and utilized an inductive qualitative design to capture the perceptions and experiences of Australian primary care dietitians about using information technology in practice [25]. Potential participants were any dietitian working in Australia who had conducted consultations with patients in the primary care setting.

### Recruitment

We used purposive and snowball sampling to recruit participants. The recruitment was conducted through direct emails to the members of various dietetic groups, including Dietitian Connection and Hunter Private Practice Dietetics Group, and through posts on dietetic Facebook groups. Participants were asked to forward recruitment materials to other potential participants within their own networks. In addition, participant characteristics (including gender, location, and years of experience) were monitored throughout the recruitment to ensure a broad range of participants, for example, both genders were included.

### Data Collection

Data were collected as part of a larger study that also explored primary care dietitians’ perceptions about the effectiveness and efficiency in the workplace. Data collection involved individual semistructured telephonic interviews of 18 and 65 (average, 48) minutes. Data collection was conducted in March 2017 and April 2017. Interviews were conducted using a semistructured interview protocol with open-ended questions that aimed to direct discussion around primary care dietitians’ perceptions and experiences about using information technology in practice. In addition, the interview protocol was developed following a literature review and discussion with a primary care dietitian about the appropriateness of questions. Questions were initially piloted with 2 primary care dietitians within the research team’s network and used to gather feedback on the flow of conversation and provide a model for subsequent interviews. In this study, only the second pilot was included because it accurately reflected the subsequent interviews. Telephonic interviews were recorded using a digital dictaphone and transcribed verbatim. All transcripts were emailed to participants to confirm the accuracy.

### Data Analysis

Data analysis was conducted alongside data collection using a thematic analysis approach. Thematic analysis consisted of reading each transcript thoroughly to identify, analyze, and report patterns in the data. Although the first 3 transcripts were analyzed in triplicate, the remainder were analyzed in duplicate. Results from analyses were then compared and combined to ensure that codes accurately reflected participants’ responses. Once the codes were confirmed, themes and subthemes were organized and assigned relevant quotes from the transcripts. In addition, triangulation was conducted with all team members whereby themes and subthemes were reviewed during regular meetings to ensure they reflected the data for transferability and credibility.

## Results

### Participants’ Characteristics

In this study, 20 primary care dietitians (n=17 females) participated in a semistructured interview. Data saturation was reached after 17 interviews because no new codes emerged from the further analysis. Participants were located across 3 states of Australia (New South Wales, Queensland, and Victoria), had a diverse range of experience in primary health care (8 months to 23 years), and most of them worked on a part-time basis. Table 1 summarizes the participants’ demographic characteristics.

From the interviews, 4 themes and 10 subthemes emerged, as displayed in Table 2; these were as follows: improving the efficiency of practice tasks, experiencing barriers to using information technology in practice, enhancing outcomes through education and monitoring, and sharing information with others. In addition, several types of technologies were discussed during interviews. The supplementary material describes their names and purpose.

### Theme 1: Information Technology Improving the Efficiency of Practice Tasks

The first theme identified several ways in which information technology could enhance the efficiency of the dietetic practice. Using information technology to aid administrative tasks was viewed as time saving, particularly for writing reports to other health care professionals. As one participant noted:

Electronic messaging...to GPs, it’s super quick. I've just got standard, self-populating letters that puts in all the basic information and then I can expand on that.

P16, 10 years’ experience

The time saved using information technology allowed dietitians to further focus on clients’ needs:

Using electronic notes...my attention is now very much on the client.

P10, 2 years’ experience

**Table 1:** The demographic characteristics of the primary care dietitians interviewed in this study.

Interview number	Interview length (min)	Gender	Years qualified as a dietitian	Years working in primary health care	Contracted hours in primary health care	Location of employment	Percentage chronic disease management referrals of total business
1	50	Female	6	1.5	Part time	NSW^a^	0
2	18	Female	4.5	4	Full-time	NSW	0
3	57	Female	6	6	Part time	NSW	90
4	52	Male	15	10	Full time	NSW	0
5	55	Female	8	8	Part time	QLD^b^	90
6	46	Female	2 or 3	0.7	Part time	VIC^c^	50
7	46	Female	5	4	Full time	VIC	85
8	45	Female	30	6	Part time	QLD	90
9	63	Female	33	15	Part time	QLD	95
10	72	Female	3	2	Part time	NSW	40
11	39	Female	3	2.5	Part time	NSW	90
12	56	Female	5	5	Part time	NSW	80
13	44	Female	15	13	Part time	QLD	<10
14	47	Female	9	9	Part time	NSW	20
15	51	Female	3	3	Full time	QLD	90
16	38	Female	25	10	Part time	QLD	90
17	37	Male	14	13	Part time	QLD	90
18	65	Male	7	7	Full time	QLD	95
19	34	Female	28	23	Part time	VIC	60
20	26	Female	29	11	Full time	VIC	20

^a^NSW: New South Wales.

^b^QLD: Queensland.

^c^VIC: Victoria.

**Table 2:** Themes and subthemes emerging from interviews.

Theme label	Theme description	Subthemes
Improving the efficiency of practice tasks	Information technology can facilitate greater efficiency in dietetic practice	Information technology aids administrative tasks; information technology aids consultation tasks
Experiencing barriers to using information technology in practice	There are numerous barriers to using information technology in the dietetic practice	Implementing new information technology is a low priority; information technology impairs communication with patients; information technology is considered expensive and unreliable
Enhancing outcomes through education and monitoring	Information technology improves patient outcomes by supporting education and tracking progress	Information technology is a valuable tool for patient education; digital tracking makes patients accountable and helps patients achieve goals; information technology increases access to nutrition information
Sharing information with others	Information technology enables sharing of information with other health professionals and with patients outside of consultations	Information technology can increase communication between health professionals; patient outcomes can be shared through digital databases

In addition, information technology was regarded as useful for improving the efficiency of consultation tasks, such as analyzing dietary data and scoring questionnaires concurrently as patients completed them. One dietitian discussed using a program called “Nutritics” to save time:

Rather than writing out a food diary for some of my patients, I am actually typing directly into this software system as they tell me what they eat and it gives me a nutritional breakdown of that food.

P5, 8 years’ experience

Moreover, participants discussed using information technology for collecting information before consultations, such as through their business website, iPads in office waiting rooms, and food tracking apps. Some participants also used information technology to initiate patient education prior to consultations, reporting that this increased the efficiency of the subsequent consultation; for example,

Clients can commence their nutrition education prior to their appointment...this saves time during the consultation. During the consultation, the dietitian can concentrate on consolidation of the education, goal setting and rapport building.

P9, 15 years’ experience

### Theme 2: Experiencing Barriers to Using Information Technology in Practice

The second theme acknowledged the common barriers experienced by dietitians to using information technology in practice. Barriers that discouraged participants from using information technology included a lack of time to implement new technology, lack of knowledge on available technologies, and a resistance to change. In the quotes below, participants divulge why these barriers discouraged the use of information technology.

I have registered for it [health kit] and I’ve looked at it...but it’s just one of those things where changing my processes...getting up to speed on it and having to watch tutorials, etc.

P3, 6 years’ experience

I haven’t really had exposure to e-health records and I don’t know how I would go about setting that up...So, it hasn’t happened...It would be me taking the initiative.

P14, 9 years’ experience

Thus, using information technology was a low priority for some participants:

It's down the list of priorities. So, it hasn’t happened

P14, 9 years’ experience

Others reported that using information technology during the consultation impaired communication with patients. Many dietitians reported having limited control over computer positioning causing them to face away from patients, which in turn led to poor body language, lack of eye contact, and reduced voice audibility. The impact of the information technology use on the voice audibility is articulated by one participant:

[The] elderly have hearing problems so we can’t face away from them and talk.

P5, 8 years’ experience

In addition, the cost was reported to be a barrier to using information technology in practice:

I think there are better systems that we could use but we can’t afford them

P5, 8 years’ experience

Moreover, the reliability of devices was reported to limit the use of information technology during consultations,

I use my laptop when I go to home visits...it runs out of battery eventually.

P15, 3 years’ experience

Furthermore, the internet connection was identified as being unreliable and “*the one that lets you down every single time* ” (P13*,* 13 years’ experience)*.*

### Theme 3: Information Technology Enhancing Outcomes Through Education and Monitoring

The third theme highlighted the potential for improved service delivery and patient outcomes using information technology. Participants identified opportunities for enhanced education using information technology, such as a mobile phone app for celiac disease and fermentable oligo-, di-, mono-saccharides and polyols (FODMAP) diets. These were regarded as important for patients and dietitians to feel confident in identifying foods that are appropriate for consumption.

[I can’t] remember every single food, and which one is high, low and medium

in FODMAPs. But [the app gave] the confidence, to show them how to do that.

P10, 2 years’ experience

The internet was identified as another valuable tool for patient education because it allowed dietitians to promptly access information during consultations and educate patients on finding healthy recipes.

If a patient is sitting across from me and I need to look something up then I can just look that up straight away. So, I can have the information on the spot.

P6, 8 months experience

Information technology was regarded as a useful means to help patients track their dietary behaviors and progress between consultations. It was identified that the tracking dietary intake helps to empower patients and facilitate better outcomes:

Clients get better outcomes when they are empowered by monitoring their own progress. It is vital that they get access to their medical progress information via this technology.

P9, 15 years’ experience

However, some participants expressed that recording behaviors on apps can be “*burdensome [to patients] because you’re having to record so much* ” (P3, 6 years’ experience). To overcome this challenge, some participants used photos to track food intake, which they considered to be more reliable than self-reported intake:

I ask them to take photos of their food if I can’t quite judge their portion size

P15, 3 years’ experience

Participants felt that mobile phone apps improved the accessibility and ease of nutritional information because they are portable. One participant discussed these benefits in terms of the FODMAP app:

Information[s] right there when they’re shopping...they just download it on their phone [FODMAP app].

P6, 8 months experience

However, many participants felt that older patients were less able to use the information technology to access information:

The main problem with technology is actually the age group of most of my clients I see a lot of people in their 60s and 70s.

P8, 6 years’ experience

### Theme 4: Information Technology for Sharing Information With Others

The fourth theme acknowledged the potential for greater information sharing using information technology. Most participants used patient management systems to acquire information about patients and communicate with other health care professionals. One participant articulated the use of information technology in enhancing communication:

It provides an avenue to communicate with the whole team, so anyone working at the medical centre can see it

P11, 2.5 years’ experience

Improvements in communication and information sharing were thought to ultimately enhance the continuity of care for patients:

The advantages are it means that the patient doesn’t feel that they’ve got to [repeat themselves]...they feel some kind of continuity

P10, 2 years’ experience

Despite these benefits to patient management systems, many participants expressed a desire for systems that are better suited to the specific information that dietitians collect during consultations:

For SGAs [Subjective Global Assessments] we have to do it on paper and then send it to head office and they scan it in and upload it

P13, 13 years’ experience

Information technology enabled participants to interact with patients outside of the formal consultation. Although some participants actively encouraged patients to email or short message service text message for additional support, others used social media for supplementary nutritional support and sharing recipe ideas or emerging dietary evidence.

[I] encourage clients to email and text me if they have questions...The closed Facebook groups [allow me to] give clients, gentle reminders and keep them up to speed with recipe ideas.

P12, 5 years’ experience

Participants acknowledged the potential for information technology to facilitate the collation of data and evaluate the effectiveness of dietetic services:

It would be beneficial in being able to determine the effectiveness of private practice dietitians

P1, 1.5 years’ experience

Moreover, participants expressed willingness to share patient outcomes utilizing a digital database but were concerned about the confidentiality of information and the ability of a digital database to integrate with current practice software:

It could be doubling up...putting my information into two databases...I don’t have time to do two things

P16, 10 years’ experience

## Discussion

This study was the first to use a qualitative methodology to explore Australian primary care dietitians’ perceptions about using information technology during practice. Dietitians in this study viewed information technology as beneficial to their practice and important for enhancing patient outcomes. Understanding these perceptions provides a direct insight into whether information technology is feasible to use in the dietetic practice and opportunities for greater integration of technology in the future.

Participants in this study regarded information technology as important for enhancing the efficiency of administrative and consultation tasks within the dietetic practice; these included administrative technology, mobile phone apps for tracking dietary intake, patient management systems, and Nutritics (a data analysis system). Technological devices have previously been shown to reduce dietitian workloads [11,13,26]; for example, using an iPad compared with paper-based forms may reduce consultation time by 5 minutes [27] and using electronic systems may reduce consultation time by 13 minutes [11]. Furthermore, an estimated 40% of preconsultation assessments can be completed remotely using information technology [27]. There are clear benefits of information technology use for improving the consultation efficiency.

Despite benefits to efficiency, better integration of dietary assessments with current health care electronic systems is needed. Interviews from this study indicate that the utilization of preconsultation data collection is not optimal with a digital assessment of the dietary intake and patient information being obtained separately. However, one participant utilized an independent app, NERO [28], which integrated the collection of dietary and medical assessments. Nevertheless, a literature review does not identify any similar app widely available. In addition, other studies acknowledge the needs of dietitians with regard to the technology design, such as linking app data with electronic health records for better work efficiency [29,30]. However, most studies investigate the app quality and patient usability [22,31] as opposed to dietitian preferences to support practice tasks. In 2013, a survey of Australian employed dietitians reported that 77% had no experience with nutrition-related information technology systems [32]. It has previously been identified that education, training, and advocacy of technology needs to be provided by dietetic associations to encourage better use or development of information technology within the profession [29,33]. Clearly, there is a need for better promotion or further development of digital assessments in health care to accurately reflect dietitians’ needs and, therefore, further enhance the efficiency.

Dietitians in this study felt that electronic patient management systems support better communication and sharing of patient information. Similar benefits of patient management systems have been previously highlighted through surveys of dietitians [5]. However, participants in this research acknowledged that these systems were not well integrated with dietitian needs. Currently, there are over 20 commercial systems available to primary care dietitians that do not align with the nutrition care process [34]. Considering that most participants reported using patient management systems in their practice, further work is needed to streamline the dietetic documentation.

Participants regarded information technology as useful for education and monitoring progress. Smartphone apps have been shown to promote self-monitoring by reducing the burden of recording [20]. In addition, the availability of barcode scanning, nutrient databases, and image recording contributes to the reduced burden [35,36]. Studies have reported that the inclusion of features in apps that are user friendly and reduce time burden is more effective [37]. A review of 23 studies showed that smartphone apps are beneficial in targeting behavioral change (17 studies) and increasing the retention rate for interventions (19 studies) [37]. In light of this, dietitians worldwide use information technology for patient self-monitoring [22,23], and dietitians in United Kingdom are recommended to use information technology to support their practice [38]. Overall, the use of information technology in the dietetic practice appears to be well accepted and encouraged internationally.

In 2014, the Academy of Nutrition and Dietetics Health Informatics Infrastructure was launched as a tool that enables documentation and standardized data collection for outcomes research [39]. The Academy of Nutrition and Dietetics Health Informatics Infrastructure system is based on the nutrition care process and international dietetic terminology [39]. This research shows that using information technology for tracking standardized data on nutrition-related outcomes could be beneficial for promoting evidence-based improvements in nutrition care. Similar benefits have been identified among other health professions, such as for identifying survival rates and characteristics among never smokers for patients with lung cancer [40]. Systems that can systematically document outcomes may be beneficial to dietitians by pooling data to evaluate the impact of structures and processes on patient outcomes. Most participants expressed interest or willingness to enter data using similar technologies. Likewise, focus group discussions assessing dietitians’ perceptions of a prototype Web-based electronic recording system indicated a possibility that it was easy to use and would be useful in patient management systems [41]. Alternate views are represented in surveys, showing that Australian dietitians lack readiness for eHealth [30]. However, survey results between 2013 and 2016 showed an improvement for eHealth readiness [30], which suggests further acceptance from the dietetic profession regarding the eHealth implementation. Therefore, this study supports trailing standardized data collection using information technology within the broader dietetic population.

There are some notable limitations to this study. First, interviews were conducted over the phone, which limited the ability to monitor body language and other interpersonal cues. However, utilizing telephonic interviews to collect data extended the geographical access of participants and allowed a broad range of individuals to be represented. Although Western Australia, Tasmania, and South Australia were not included in the study sample, this reflects the higher proportion of employed dietitians within the other states of Australia [42]. Second, the interviews were conducted by 3 interviewees, which may have reduced the consistency among interviews. However, all interviewees were concurrently trained in the interview protocol and were present at each interview, enabling prompting, which promoted consistency among participants.

Information technology appears to be an important and acceptable component of the dietetic practice in primary care. This study adds insight into the variety of ways in which information technology can benefit dietitians, while also identifying several barriers to overcome for its optimal use. The potential benefits of information technology include better communication and information sharing among health care professionals to support patient outcomes, improved access to information to support education and interventions, digital dietary tracking to encourage patient accountability and better outcomes, better communication and accessibility of dietetic services (ie, Facebook groups); and potential to support improvements in nutrition care through standardized databases and outcomes research.

The opportunities for improved patient outcomes through information technology use warrant strategies to support dietitians overcome barriers to integrating technology into practice. These barriers include technology detracting from the patient-dietitian communication, technology not being appropriate for certain patient groups, or tasks and technology not integrating well into dietetics. It is important that employers support dietitians by providing training on effectively using technology in consultations or setting up consultation rooms to support better body language. Technology is not suitable for some patient populations; therefore, dietitians should also be mindful of the alternative methods of educating and collecting data from these individuals. To accurately reflect dietitians’ needs, relevant nutrition resources, such as subjective global assessments and the nutrition care process, need to be included into patient management systems. Furthermore, linking app data with electronic health records or patient management systems is needed. The successful development and adoption of such information technologies will require support from dietitians and national bodies, such as the Dietitian Association of Australia and the Australian Government. Further development of information technology is needed to better support dietitians in the workplace.

## Appendix

Multimedia Appendix 1 A descriptive list of the technologies dietitians use to aid their practice.

## Abbreviations

FODMAP: fermentable oligo-, di-, monosaccharides and polyols
