# Supplementary material for: Investigating the Perceptions of Primary Care Dietitians on the Potential for Information Technology in the Workplace: Qualitative Study
Source: J Med Internet Res. 2018 Oct 15;20(10):e265. doi: 10.2196/jmir.9568 (PMC6231874; doi:10.2196/jmir.9568)
Supplement: Multimedia Appendix 1 [file jmir_v20i10e265_app1.pdf]

A descriptive list of the technologies dietitians use to aid their practice

Name	Purpose
**Administrative**	
Xero	An online accounting Software
Google Keep	An online note taking service – written, audio, and visual. Sets reminders
Appointment Reminder	An online system that integrates with existing software, such as google calendar and outlook calendar, to sends customised text message and email appointment reminders to clients
EFTPOS Machine	Electronic funds transferred at point of service
HICAPS	A digital service for health industry claims and payments services. Supports 98% of health funds, delivering a simple health claim billing solution for healthcare providers
PRODA (provider digital access)	An online software that provides secure access to specific government services, including Health Professional Online Services and the National Disability Insurance Scheme
Power Diary	A software suited for smartphones and desktop computers that is a secure calendar system for bookings. Records patient information and tracks visits. Supports Medicare claiming online. Sends SMS or email reminders. Creates templates for commonly used correspondence (i.e. referral acknowledgement, progress notes), which can be personalised by merging fields. Handles billing and paying, creates reports to monitor business, health industry compliant, integrated with Xero
**Practice Management Software**	
Best Practice Software	An online software with integrated clinical and practice management modules. Billing payments supported. Remote access
HealthKit	An online software with invoices and payments, patient records, financial reports, directory and online bookings
myPractice	An online software for appointment management with SMS or email reminders, calendar, patient notes, financial details
MediFlex	An online software for Medical Accounting, Practice Management and Clinical Software
Medical Director	An online software that manages reports and referrals online. Information sharing. Online access to health care tools such as, textbooks and journals. Provides drug interaction information, calculators and charts
**Data Collection**	
Easy Diet Diary	Smartphone app to track food intake by scanning barcodes, searching food database or taking photos. Tracks energy intake, nutrients, energy burned through exercise and weight. Potential to connect and share information with dietitians
My Diet Dairy Calorie Counter	Smartphone app to track food intake by searching food databases. Tracks energy intake, nutrients, energy burned through exercise and weight. Connects and integrates with apps (i.e. Fitbit)
mySymptoms Food & Symptoms Tracker	Smartphone app to track food intake and intensity of symptoms experienced. Analysis algorithms to determine patterns between diet and symptoms
Medibank Energy Balancer	A smartphone app that balances food intake and exercise
My Fitness Pal	A software suited for smartphones and computer desktops to track food intake by searching food database. Tracks energy intake, nutrients, steps, energy burned through exercise and weight. Connects and integrates with apps (i.e. Fitbit)

Name	Purpose
NERO (nutrition education resources online)	A software for smartphones and computer desktops to support the nutritional professional and customer to achieve better health outcomes. Includes educational videos and nutritional assessment
Nudge	A software for smartphones and desktop computers that organises data from leading apps and wearables to help better monitor progress towards health goals. Ability to work with a health coach to help identify which health habits need improving most
iPhone Health App - Dashboard	A smartphone application that tracks steps or distance walking/running over a day, week, month or year
Camera	A hardware component of phones used to take photos and record food intake
Fitbit	Wearable device that tracks activity, exercise, weight and sleep via a smartphone app.
**Education**	
Monash University Low FODMAP Diet	A smartphone appl that includes a food guide detailing FODMAP content, recipe book, shopping list for organising low FODMAP purchases, one week food and symptom journal (ability to view graphs of symptoms)
FODMAP Friendly	A smartphone app that includes food guide detailing FODMAP content, and FODMAP Friendly approved foods
Gluten Free Eating Directory	Database of Australian gluten free restaurants, shops and manufactures documented on a smartphone app or desktop computer
Coeliac Australia	A smartphone app that includes an ingredients list detailing over 800 ingredients and 300 additives used in Australia and New Zealand foods and indicates their suitability for inclusion into a gluten free diet
Calorie King	A software for smartphones or desktop computers that has a food database with information on Australian foods energy, fat, carbs, protein, fibre, saturated fat, trans fat, cholesterol, sodium and alcohol
NERO	*See above
RapidCalc Diabetes Manager	A smartphone app with a bolus dose calculator for diabetes following a basal-bolus regime. Intelligent tracking of insulin on board, automatic carbohydrate ratio and blood glucose target selection. Includes history, charting and statistics features
Food Switch	A smartphone app which scans foods and offers healthier or more suitable alternatives. Includes SaltSwitch, GlutenSwitch, FatSwitch, EnergySwitch, SugarSwitch and FoodSwitch Classic
Headspace	A smartphone app for guided meditation and mindfulness
ReachOut Breathe	A smartphone app to reduce physical symptoms of stress and anxiety by slowing down your breathing and heart rate
**Data Analysis**	
mySymptoms Food & Symptoms Tracker	*See above
Nutritics	Cloud-based software database used for nutritional analysis in real-time as a food dairy is being reordered. Menu planning tool, and recipe analysis tool
FoodWorks	An online software database for Australian food for nutritional analysis
Online Australian Eating Survey	An online software for food frequency questionnaires that provides immediate analysis of results compared to food and nutrient targets, personalised to age, gender and life stage

Name	Purpose
**Storage**	
Customer Relationship Management (CRM) Software	A software for smartphones and desktop computers that analyses and stores customer interactions and data throughout a customers' lifecycle. The software also automates workflow processes, including tasks, calendar and alerts. The purpose of the software is to provide businesses the ability to track performance and productivity and improve customer relationships, customer retention and sales growth
Dropbox	A software for smartphones and desktop computers with secure file storage, sharing, collaboration
Google Keep	*See above
**Communication**	
Sky Clinic	Internet portal for health care professionals to share knowledge, resources, documents, referrals, ideas and communicate
Zoom	Video conferencing and web conferencing service, delivered via a smartphone app or desktop computer
Skype	Provides instant online text messages and video chat, delivered via a smartphone app or desktop computer
Facebook	Social media and social networking service, available on a smartphone app or desktop computer
